# Cytokine Profile of CCR6^+^ T-Helpers Isolated from the Blood of Patients with Peptic Ulcer Associated with *Helicobacter pylori* Infection

**DOI:** 10.17691/stm2020.12.3.04

**Published:** 2020-06-28

**Authors:** V.Yu. Talayev, M.V. Svetlova, I.E. Zaichenko, E.V. Voronina, O.N. Babaykina, N.V. Neumoina, K.M. Perfilova, O.V. Utkin, E.N. Filatova

**Affiliations:** Professor, Head of the Laboratory of Cellular Immunology; Academician I.N. Blokhina Nizhny Novgorod Scientific Research Institute of Epidemiology and Microbiology of Rospotrebnadzor (Russian Federal Consumer Rights Protection and Human Health Control Service), 71 Malaya Yamskaya St., Nizhny Novgorod, 603950, Russia; Senior Researcher, Laboratory of Cellular Immunology; Academician I.N. Blokhina Nizhny Novgorod Scientific Research Institute of Epidemiology and Microbiology of Rospotrebnadzor (Russian Federal Consumer Rights Protection and Human Health Control Service), 71 Malaya Yamskaya St., Nizhny Novgorod, 603950, Russia; Leading Researcher, Laboratory of Cellular Immunology; Academician I.N. Blokhina Nizhny Novgorod Scientific Research Institute of Epidemiology and Microbiology of Rospotrebnadzor (Russian Federal Consumer Rights Protection and Human Health Control Service), 71 Malaya Yamskaya St., Nizhny Novgorod, 603950, Russia; Researcher, Laboratory of Cellular Immunology; Academician I.N. Blokhina Nizhny Novgorod Scientific Research Institute of Epidemiology and Microbiology of Rospotrebnadzor (Russian Federal Consumer Rights Protection and Human Health Control Service), 71 Malaya Yamskaya St., Nizhny Novgorod, 603950, Russia; Senior Researcher, Laboratory of Cellular Immunology; Academician I.N. Blokhina Nizhny Novgorod Scientific Research Institute of Epidemiology and Microbiology of Rospotrebnadzor (Russian Federal Consumer Rights Protection and Human Health Control Service), 71 Malaya Yamskaya St., Nizhny Novgorod, 603950, Russia; Chief Physician, Clinic of Infectious Diseases; Academician I.N. Blokhina Nizhny Novgorod Scientific Research Institute of Epidemiology and Microbiology of Rospotrebnadzor (Russian Federal Consumer Rights Protection and Human Health Control Service), 71 Malaya Yamskaya St., Nizhny Novgorod, 603950, Russia; Deputy Chief Physician, Clinic of Infectious Diseases; Academician I.N. Blokhina Nizhny Novgorod Scientific Research Institute of Epidemiology and Microbiology of Rospotrebnadzor (Russian Federal Consumer Rights Protection and Human Health Control Service), 71 Malaya Yamskaya St., Nizhny Novgorod, 603950, Russia; Head of the Laboratory of Molecular Biology and Biotechnology; Academician I.N. Blokhina Nizhny Novgorod Scientific Research Institute of Epidemiology and Microbiology of Rospotrebnadzor (Russian Federal Consumer Rights Protection and Human Health Control Service), 71 Malaya Yamskaya St., Nizhny Novgorod, 603950, Russia; Leading Researcher, Laboratory of Molecular Biology and Biotechnology Academician I.N. Blokhina Nizhny Novgorod Scientific Research Institute of Epidemiology and Microbiology of Rospotrebnadzor (Russian Federal Consumer Rights Protection and Human Health Control Service), 71 Malaya Yamskaya St., Nizhny Novgorod, 603950, Russia

**Keywords:** T-helper lymphocytes, CCR6, immuno-magnetic separation, chemokine receptors, cytokines, gastric and duodenal peptic ulcer.

## Abstract

We previously found that the number of CCR6^+^ T-helpers with the phenotype of effector/effector memory T cells increases in the blood of patients with *H. pylori*-associated peptic ulcer. The mature phenotype and the expression of the chemokine receptor CCR6, which is involved in migration of lymphocytes to the inflamed mucous membrane of the gastrointestinal tract, suggests that these cells are involved in the immune response observed in this clinical condition. To better understand the pathogenetic role of these cells, it is necessary to study their functional activity, specifically, the production of pro-inflammatory cytokines involved in the pathogenesis of the disease.

**The aim of the study** was to evaluate changes in the blood level of pro-inflammatory types of mature CCR6^+^ T-helpers in *H. pylori*-associated peptic ulcer disease.

**Materials and Methods.:**

CCR6^+^ T-helpers were isolated from the blood by using immuno-magnetic separation adapted to this study. The number of T-helpers of types 1 and 17 (Th1 and Th17) and cells with mixed properties of Th1 and Th17 (Th1/Th17) was determined by intracellular cytokine assay.

**Results.:**

Initially, we planned to activate unseparated peripheral blood mononuclear cells *ex vivo* and evaluate the number of cytokine producers among mature CCR6^+^ T-helper cells by gating them during the flow cytometry. However, dramatic changes in the phenotype of T-helpers upon activation did not allow us to reliably identify the cells of interest*.* Subsequently, we used a two-stage immunomagnetic separation procedure to obtain functionally active mature CCR6^+^ T-helpers with a purity of >90%. The quantitative yield of these cells from the blood of patients with gastric and duodenal peptic ulcer associated with *H. pylori* was 9 times higher than that from the blood of healthy donors. Activation of CCR6^+^ T-helpers purified from blood of ulcer patients revealed an increased content of Th1, Th17, and Th1/Th17. One ml of the patient’s blood yielded 18.1 times more CCR6^+^ Th1, 19.4 times more CCR6^+^ Th17, and 21.1 times more CCR6^+^ Th1/Th17 compared with the blood of healthy subjects.

**Conclusion.:**

The content of mature CCR6^+^ T-helper cells with pro-inflammatory activity significantly increases in the blood of patients with peptic ulcer associated with *H. pylori* infection.

## Introduction

In this work, we studied CCR6^+^ T-helpers that were shown to increase in numbers in the blood of patients with peptic ulcer associated with *H. pylori* infection [1]. *H. pylori* is able to persist for a long time in the stomach and areas of gastric metaplasia in the duodenum, causing various types of infection, from asymptomatic to type B gastritis and peptic ulcer. Moreover, *H. pylori* infection can provoke the development of gastric adenocarcinoma and MALT lymphoma [2–4].

The immune response to *H. pylori* infection develops rapidly [5, 6], but this reaction is not always able to effectively fight the pathogen. Apparently, the most important mechanism for evading this bacteria from immune attacks is populating specific niches [7, 8], which are practically inaccessible to immunity effectors. The weakness of trans-epithelial transport of IgA in the stomach [9] does not allow creating a concentration of secretory IgA sufficient to eliminate *H. pylori* from the gastric mucus and the surface of the epithelium [10–12]. The cellular immune response to *H. pylori* also has a limited protective potential. Moreover, experiments on mice suggest that the cellular immune response, namely the activity of pro-inflammatory T-helpers, makes an important contribution to the development of gastritis symptoms [12], along with the direct effect of *H. pylori* toxins. In the absence of fully functioning T-helpers, *H. pylori* causes a massive but asymptomatic infection in mice, while restoring immunity in these mice by the introduction of normal T-helpers causes a decrease in the bacterial load and, at the same time, a severe inflammation of the gastric mucosa [13, 14]. The main cytokines that induce inflammation in *H. pylori*-associated gastritis models are the Th1 cytokine interferon-g (IFN-g) and the Th17 cytokine interleukin-17A (IL-17A) [13]. Notably, the maturation of Th17 and their production of IL-17A are crucial for the secretion of IFN-g [15, 16]. The contribution of immune mechanisms to the formation of peptic ulcer has not been adequately studied.

Previously [1], we found that in the blood of patients with gastric and duodenal peptic ulcer associated with *H. pylori*, the number of mature T-helpers expressing the chemokine receptor CCR6 was twice higher than normal, while in gastroduodenitis, the number of these cells increased insignificantly. The CCR6 directs the migration of T-lymphocytes from the bloodstream to the skin, Peyer’s plaques, as well as to the inflamed mucous membrane of the gastrointestinal tract [17, 18]. The expression of CCR6 is characteristic of Th17, although the number of CCR6^+^ T-helpers significantly exceeds the number of mature Th17 [19]. In our opinion, the increase in the number of mature CCR6^+^ T-helpers occurs because of their involvement in the immune response to *H. pylori*. We suggest that having left the secondary lymphoid organs after maturation and once in the bloodstream, these cells can migrate into the inflamed mucosa, where they will produce cytokines in accordance with the cytokine profile that they acquired during maturation. In this work we determine the number of mature Th1, Th17, and Th1/Th17 in CCR6^+^ T-helpers separated from the blood of healthy donors and ulcer patients.

**The aim of the study** was to evaluate changes in the blood level of pro-inflammatory types of mature CCR6^+^ T-helpers in *H. pylori*-associated peptic ulcer disease.

## Materials and Methods

The study was conducted in accordance with the provisions of the Helsinki Declaration (2013) and approved by the local Ethics Committee of the Academician I.N. Blokhina Nizhny Novgorod Scientific Research Institute of Epidemiology and Microbiology. All patients and control subjects gave their informed consent to participate in the study. Adult patients (n=7) with gastric or duodenal ulcer complained of an exacerbation of the underlying disease and were examined upon admission to the hospital. The presence of *H. pylori* was confirmed using a quick urease test and a *H. pylori* DNA test with the polymerase chain reaction [20]. Adult healthy controls were enrolled in the study as a group of comparison (n=15) and for testing the methodology (n=4).

### Activation of unfractionated peripheral blood mononuclear cells (PBMCs).

PBMCs were isolated from heparinized venous blood in the traditional way by centrifugation over a Hystopaque-1077 layer (Sigma, USA). Then, cells were seeded in 24-well plates (Costar, USA) at a density of 2**·**10^6^ cells per well in 1 ml of RPMI-1640 medium (Gibco, UK) with 10% fetal calf serum — FCS (PAA Laboratories, Austria) and activators: 25 ng/ml phorbol-12-myristate-13-acetate — PMA (Serva, Germany) and 1 μg/ml ionomycin (Sigma). Cells were cultured for 4 or 22 h at +37°C and 5% CO_2_; in both versions, 5 μg/ml brefeldin A (BioLegend, USA) was added for 4 final hours of cultivation. Then, the cells were stained with fluorescent conjugates of antibodies to the molecules of CD4 (Sorbent, Russia), CD45RO, CD196 (CCR6) (BioLegend), and CD197 (CCR7) (eBioscience, USA) and fixated with 4% paraformaldehyde. The membranes were permeabilized with 0.1% saponin followed by cell staining for cytokines using conjugates of antibodies to IFN-g and IL-17A (eBioscience), as described previously [21]. The samples were analyzed using a flow cytometer FacsCanto II (BD Biosciences, USA), aiming at evaluating the number of IFN-g and IL-17A producers in CD4^+^, CD4^+^CD45RO^+^, and CD4^+^CD45RO^+^CSR6^+^CCR7^–^ cells.

### Immuno-magnetic separation of CCR6^+^ T-helper cells.

At the first stage of separation, CD4^+^ T-helpers were isolated from PBMCs by negative selection using the EasySep Human CD4^+^ T-Cell Isolation Kit (STEMCELL Technologies, Canada) according to the manufacturer’s instructions. At the second stage, CCR6^+^ cells were isolated from the T-helper cell fraction by positive selection using individual reagents from STEMCELL Technologies. To this end, T-helpers isolated from 18 ml of blood were placed into a round-bottom polystyrene tube (12 mm wide and 75 mm long), washed once, and resuspended in 350 μl of a magnetic bead medium (MBM) composed of phosphate-buffered saline, 2% FCS and 1 mM EDTA. Then, 9 μl of the EasySep Human CCR6^+^ Positive Selection Cocktail II reagent (STEMCELL Technologies) was added to link the CCR6 to the bead surface. After 5 min of gentle stirring at room temperature, 70 μl of EasySep Releasable RapidSpheres 50201 magnetic bead suspension was added to the cells and resulting suspension was incubated for another 5 min. The volume was adjusted to 2.5 ml with MBM, mixed by gentle pipetting and placed in an EasySep magnet. After 5 min, the liquid was drained from the tube without removing the tube from the magnet. Then the tube was withdrawn from the magnet and the remaining cells in it were resuspended in 2.5 ml of MBM. The tube was again placed in the magnet, and after 5 min, the liquid was again drained without removing the tube from the magnet. The tube was taken out and cells were washed with 175 μl of MBM from wall of the tube. Then, 35 μl of EasySep Release Buffer was added followed by incubation for 5 min. The tube was again placed in the magnet. After 5 min, the fraction containing CCR6^+^ T-helpers was withdrawn and transferred into a fresh sterile tube. The purity of the final product and the samples taken during the purification stages were determined by flow cytometry after the cells were stained with conjugates of antibodies to CD4, CD45RO, and CCR6.

### Activation of purified T-helpers.

The purified cells were washed and seeded in round-bottom 96-well plates (Costar), 2.5**·**10^4^ cells in 100 μl of RPMI-1640 medium with 10% FCS. Cells were cultured from 22 to 118 h at +37°C and 5% CO_2_. Activation was carried out by adding 25 ng/ml PMA and 1 μg/ml ionomycin for the final 4 or 22 h of cultivation. Brefeldin A (5 μg/ml) was added for the final 4 h of cultivation. After cultivation, cells were harvested and stained for intracellular cytokines as described above and analyzed by flow cytometry.

### Statistical data processing.

Cytometry results were processed by BD FACSDiva software (BD Biosciences). Statistical analysis was performed using the Student t-test.

## Results and Discussion

**Figure 1:**
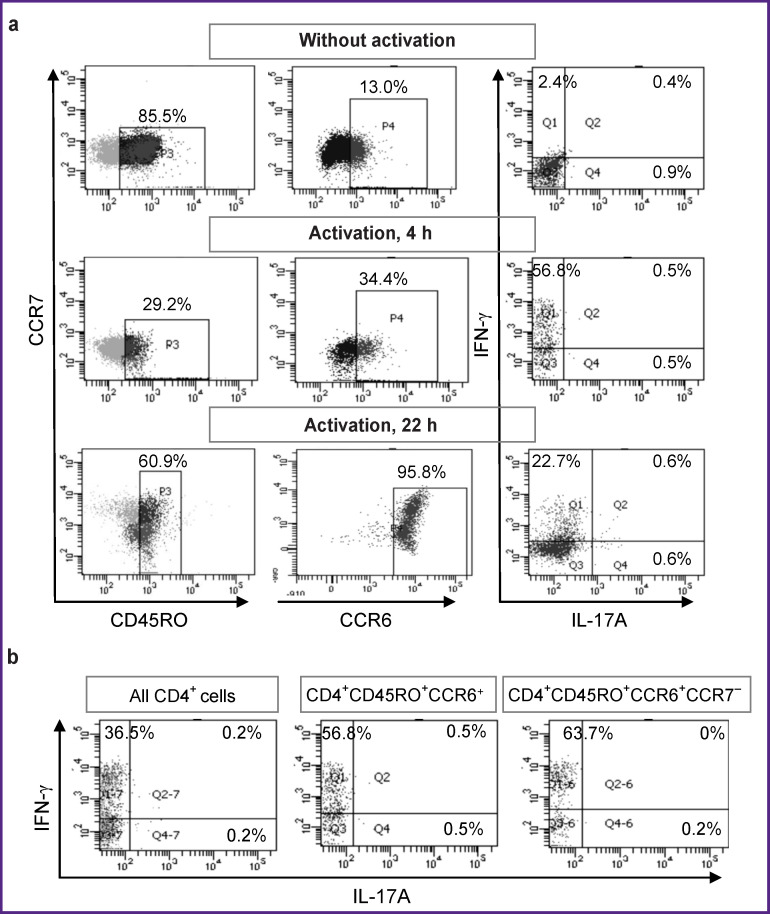
Change in the phenotype of CD4^+^ T-helpers after activation unseparated PBMCs: (a) PBMCs were cultured without activation or activated with PMA and ionomycin for 4 or 22 h. T-helpers were gated as CD4^+^ lymphocytes. From composition of this subpopulation, CD45RO^+^ T-helpers were selected in gate P3 and CCR6^+^ T-helpers were selected in gate P4. The numbers of gated cells are shown as percentages of all T-helpers. The expression of IFN-g and IL-17A in CD45RO^+^ T-helper cells is shown in the right columns of dotplots. The proportion of IFN-g^+^IL-17А^–^ cells (%) is indicated in the Q1 quadrants, that of IFN-g^+^IL-17A^+^ — in Q2, and that of IFN-g^–^IL-17A^+^ — in Q4; (b) expression of IFN-g and IL-17A upon 4-hour activation of PBMCs and subsequent gating of T-helper cells of different phenotypes (phenotypes indicated above the graphs)

Initially, we planned to activate unseparated PBMCs, and then evaluate the proportion of IFN-g and IL-17A producers among CD4^+^CD45RO^+^CСR6^+^CCR7^–^ T-helper cells by gating them during the flow cytometry analysis. However, we were compelled to abandon this plan after we found significant changes in the cell phenotype upon activation. Activation of PBMCs during 4 h induced a decrease in CD4 and CD45RO expression, as well as a marked increase in CCR6 expression (Figure 1 (a)). After 22 h of activation, CCR6 expression increased many times, as a result of which almost all T-helpers acquired this molecule. These strong phenotypic changes did not allow us to single out the object of our study, namely, the small group of mature CСR6^+^ Т-helpers present in the initial blood samples. Instead, using the flow cytometry gating option, we isolated a large number of T-helpers, initially lacking CCR6 but expressing it during activation. Regarding the production of IFN-g and IL-17A, these cells did not differ from the main group of mature CD4^+^CD45RO^+^ T-helpers: most of the cytokine producers were IFN-g^+^ Th1, while the number of IL-17A producers was negligible (Figure 1 (b)).

**Figure 2:**
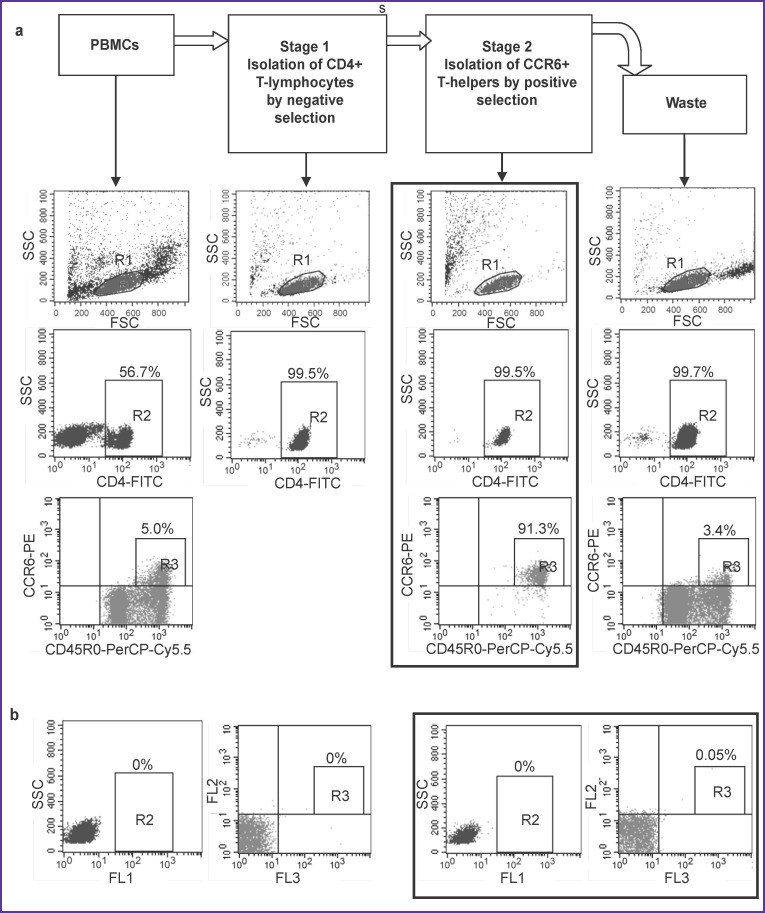
Separation of CCR6^+^ T-helpers: (a) phenotypes of cells obtained at the stages of purification; phenotype of the final purification product — mature CCR6^+^ T-helpers — is framed; (b) staining control of PBMCs and final product of purification (in the frame). The cells are sequentially gated: lymphocytes — in gate R1, then CD4^+^ lymphocytes — in gate R2, then CD4^+^CD45RO^+^CCR6^+^ lymphocytes — in gate R3. The quantity of gated cells is indicated as percent of the total number of lymphocytes

As a result, we abandoned the initial idea to activate unfractionated PBMCs and decided to isolate the target group of cells using magnetic separation and only then activate them. Since there are no commercial kits for the isolation of mature CCR6^+^ T-helper cells, we had to modify the purification method using the CD4^+^ T-helper kit and individual reagents from the Th17 enrichment kit. We used a purification procedure as follows. At the first stage of separation, CD4^+^ T-helpers were isolated from PBMCs by negative selection. In this case, all cells other than T-helpers were bound to magnetic beads with antibodies to linear markers and then separated in the magnetic field. The bead-free T-helpers were collected and used in the next separation step. At the second stage, CCR6^+^ cells were linked to magnetic beads by the respective antibodies and then separated from other T-helper cells in the magnetic chamber. Then we detached the magnetic beads from the cell surfaces, removed beads by magnet and got the final product i.e., CCR6^+^ T-helpers. Phenotypic analysis showed that this method was able to efficiently isolate a group of T-helpers with a high level of CCR6 expression, while the waste fraction contained CCR6^–^ T-helpers and a residual amount of T-helpers with a low level of CCR6 expression (Figure 2). Since only mature T-helpers were shown to express high levels of CCR6 [1], the final separation product contained largely pure fraction of mature cells with the phenotype of CD4^+^CD45RO^+^CCR6^+^. Purity of CD4^+^ cells after the first separation step was greater than 99%, the purity of CD4^+^CD45RO^+^CCR6^+^ cells after the second separation exceeded 90% (see Figure 2). Thus, the cells isolated by this method represented the object of this study — circulating mature CCR6^+^ T-helper cells.

**Figure 3:**
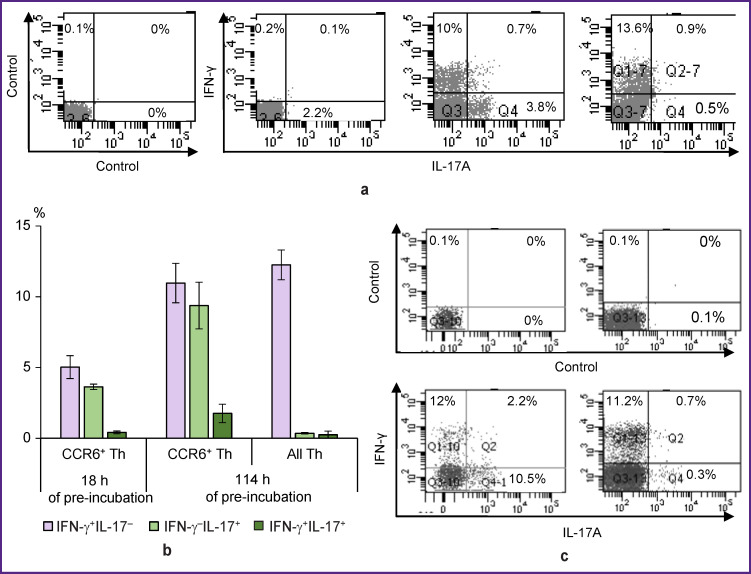
Expression of IFN-γ and IL-17A in mature CCR6^+^ T-helper cells isolated from the blood of healthy subjects: (a) from left to right: staining control; expression of IFN-γ and IL-17A in cells cultured for 4 h without activation; activation for 4 h; activation for 22 h; (b) proportion of cells producing IFN-γ, IL-17A, and both cytokines simultaneously, in cultures of mature CCR6^+^ T-helpers (CCR6^+^ Th) and unseparated T-helpers (all Th) activated within 4 h after 18 or 114 h of pre-incubation; (c) expression of IFN-γ and IL-17A in mature CCR6^+^ T-helpers (*left pair of graphs*) and unseparated T-helpers (*right pair of graphs*) after 114 h-pre-incubation and 4 h-activation. Top panel_s_: staining control

To develop a method for assessing the functional properties of the final cell fraction, purified CCR6^+^ T-helpers from healthy subjects were activated with a mixture of PMA and ionomycin for 4 or 22 h. Cells without activators served as a control. In order to accumulate the produced cytokines inside the cells, the intracellular transport inhibitor, brefeldin A, was added to all cultures for the last 4 h of cultivation. Staining for IFN-g and IL-17A showed that non-activated CCR6^+^ T-helper cells did not produce these cytokines (Figure 3 (a)). Short-term activation during 4 h caused the accumulation of IFN-g and IL-17A in two different cell groups — Th1 and Th17, respectively. We also detected a small number of cells with mixed properties — Th1/Th17. After 22 h-activation, the number of detected Th1 cells changed insignificantly compared to short-term activation, while Th17 cells almost ceased to be detected. It can be suggested that mature circulated CCR6^+^ Th17 cells rapidly begin producing IL-17A upon activation but stop this production within 18 h, whereas CCR6^+^ Th1 keep their IFN-g production throughout the full cultivation period.

Non-activated purified CCR6^+^ T-helper cells were able to support their functional properties during prolonged cultivation. We cultivated these cells in a medium without activators for 18 or 114 h, and then activated them for 4 h in the presence of brefeldin A. After 18-hour pre-incubation, a Th1 and Th17 were found in about identical numbers but Th1/Th17 were almost absent (Figure 3 (b)). After 114-hour pre-incubation, the number of cells identified as Th1, Th17, and Th1/Th17 did not decrease and even tended to increase. CD4^+^ T-helpers, which were not divided into CCR6е^+^ and CCR6^–^ subsets, under similar activation conditions, mainly demonstrated the properties of Th1 (Figure 3 (b), (c)).

**Figure 4:**
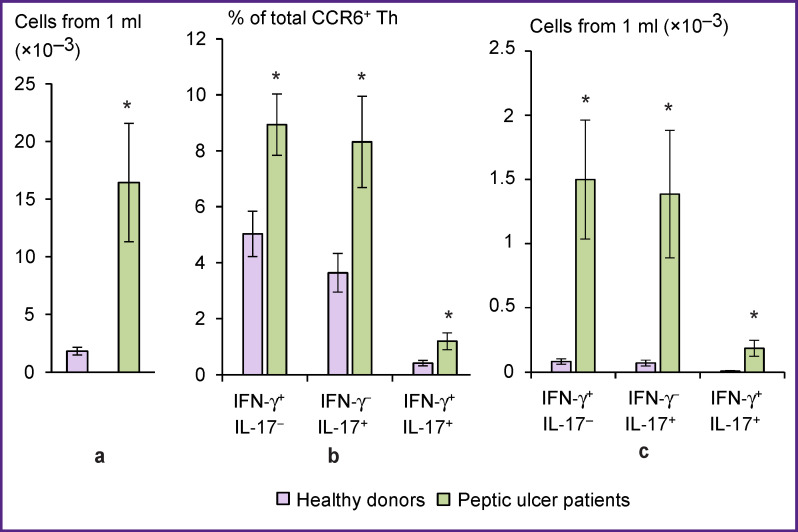
Yield of mature CCR6^+^ T-helpers from the blood of healthy donors (n=15) and patients with peptic ulcer disease (n=7); cytokine production by these cells: (a) yield of mature CCR6^+^ T-helpers from 1 ml of blood; (b) proportion of cytokine producers in cultures of mature CCR6^+^ T-helper; (c) yield of cytokine-producing mature CCR6^+^ T-helpers from 1 ml of blood. The combination of produced cytokines is shown at the bottom; * significantly different from healthy donors (p<0.05)

To compare CCR6^+^ T-helpers in the blood of peptic ulcer patients with those of healthy controls, we isolated the cells by magnetic separation, then pre-incubated them for 18 h and activated with PMA and ionomycin in the presence of brefeldin A for 4 h. The yield of purified mature CCR6^+^ T-helpers in healthy donors was 0.13±0.02% of the initial number of PBMCs. In patients with peptic ulcer disease, the output of purified cells reached 1.04±0.31% (p=0.02). The number of mature CCR6^+^ T-helpers isolated from 1 ml of blood of peptic ulcer patients, was 9 times higher than that of controls (Figure 4 (a)). CCR6^+^ T-helper cells isolated from the patients contained significantly higher proportions of Th1, Th17, and Th1/Th17 in comparison with subjects in the control group (Figure 4 (b)). The quantitative yield of pro-inflammatory T-helper cells from 1 ml of patient’s blood was 18.1 times greater for CCR6^+^ Тh1 (р=0.02), 19.4 times greater for CCR6^+^ Тh17 (p=0.03) and 21.1 times greater for CCR6^+^ Th1/Th17 (p=0.03) as compared with the blood of healthy subjects (Figure 4 (c)).

## Conclusion

Using immuno-magnetic separation, we obtained functionally active mature CCR6^+^ T-helper cells with a purity of >90% from samples of peripheral blood. The yield of these cells from blood of patients with *H. pylori*-associated peptic ulcer was 9 times higher than in healthy donors. Activation of purified CCR6^+^ T-helper cells *ex vivo* revealed an increased content of Th1, Th17, and Th1/Th17 in patients with peptic ulcer disease. The increase in the number of mature CCR6^+^ T-helper blood cells and their increased pro-inflammatory activity suggest the pathogenetic significance of these cells in *H. pylori*-associated peptic ulcer disease.
